# RBM24 stabilizes hepatitis B virus pregenomic RNA but inhibits core protein translation by targeting the terminal redundancy sequence

**DOI:** 10.1038/s41426-018-0091-4

**Published:** 2018-05-14

**Authors:** Yongxuan Yao, Bo Yang, Huang Cao, Kaitao Zhao, Yifei Yuan, Yingshan Chen, Zhenhua Zhang, Yun Wang, Rongjuan Pei, Jizheng Chen, Xue Hu, Yuan Zhou, Mengji Lu, Chunchen Wu, Xinwen Chen

**Affiliations:** 1 State Key Laboratory of Virology, Wuhan Institute of Virology, Chinese Academy of Sciences, Wuhan, China; 2 University of Chinese Academy of Sciences, Beijing, China; 3 Department of Infectious Diseases, The First Affiliated Hospital, Anhui Medical University, Hefei, 230022 China; 4 School of Pharmacy, Anhui Medical University, Hefei, 230022 China; 5 Institute of Virology, University Hospital of Essen, Essen, Germany

## Abstract

The terminal redundancy (TR) sequence of the 3.5-kb hepatitis B virus (HBV) RNA contains sites that govern many crucial functions in the viral life cycle, including polyadenylation, translation, RNA packaging, and DNA synthesis. In the present study, RNA-binding motif protein 24 (RBM24) is shown to be involved in the modulation of HBV replication by targeting the TR of HBV RNA. In HBV-transfected hepatoma cell lines, both knockdown and overexpression of RBM24 led to decreased HBV replication and transcription. Ectopic expression of RBM24 inhibited HBV replication, which was partly restored by knockdown of RBM24, indicating that a proper level of RBM24 was required for HBV replication. The regulation of RBM24 of HBV replication and translation was achieved by the interaction between the RNA-binding domains of RBM24 and both the 5′ and 3′ TR of 3.5-kb RNA. RBM24 interacted with the 5′ TR of HBV pregenomic RNA (pgRNA) to block 80S ribosome assembly on HBV pgRNA and thus inhibited core protein translation, whereas the interaction between RBM24 and the 3′ TR enhanced the stability of HBV RNA. Finally, the regulatory function of RBM24 on HBV replication was further confirmed in a HBV infection model. In conclusion, the present study demonstrates the dual functions of RBM24 by interacting with different TRs of viral RNA and reveals that RBM24 is an important host gene for HBV replication.

## Introduction

Hepatitis B virus (HBV) is a noncytopathic, hepatotropic virus belonging to the *Hepadnaviridae* family with a partially double-stranded, relaxed circular DNA genome of 3.2 kb. Transcription of the HBV genome produces four major mRNAs, including the 3.5-kb pregenomic RNA (pgRNA)/preC RNA and 2.4-kb, 2.1-kb, and 0.7-kb subgenomic RNA^1^. The pgRNA encodes both the polymerase and core protein and also serves as the template for HBV DNA replication, thus playing an essential role in HBV replication^2^. The preCore RNA encodes the preCore protein, which is post-translationally processed to become the mature HBV e-antigen (HBeAg)^2^. The 2.4-kb RNA and 2.1-kb RNA encode HBV large surface protein (LHBs), middle surface protein (MHBs), and small surface protein (SHBs), respectively^1^.

HBV RNA has variable 5′ ends, modified by the addition of 5′ caps, which are determined by the location of the core, preS1, preS2, and X promoters, but terminate at a common 3′ end and are modified by a 3′ polyadenylation (poly (A)) signal. The 3.5-kb HBV RNA encompasses the genome length, with terminally redundant (TR) 5′ and 3′ ends. The TR sequence contains all or nearly all the precore region plus approximately 50 nucleotides of the core gene. Although the 2.4-kb, 2.1-kb, and 0.7-kb subgenomic RNA are not TR, they share a 3′ copy of the TR sequence with the 3.5-kb RNA^3, 4^. In addition to transcriptional regulation by the promoter elements and two enhancer regions (EN1 and EN2)^5, 6^, the four HBV transcripts are also modulated by some host factors at the transcriptional or post-transcriptional level. In addition to previously discovered liver-specific and ubiquitous transcription factors^7, 8^, several host factors have been reported to be involved in the post-transcriptional control of HBV transcripts. For example, a multifunctional RNA-binding protein (RBP), the La protein, stabilizes HBV RNA by interacting with a small *cis*-acting element located within the viral RNA between nucleotides (nts) 1275 and 1291^9–11^. The polypyrimidine tract binding protein binds to the HBV post-transcriptional regulatory element (PRE) and thereby increases PRE-dependent gene expression^12^. Understanding the mechanism by which HBV transcripts are modulated should provide insights into critical events that are required for its replication and the development of new antiviral targets.

RBPs, which bind to double-stranded or single-stranded RNA, play key roles in the post-transcriptional regulation of RNA. It has been reported that host RBPs can be subverted for viral replication^13^. RBP 24 (RBM24) is required for cardiovascular development and myogenesis by regulating the stability and/or alternative splicing of the messenger RNA (mRNA) of related genes^14, 15^. RBM24 contains a conserved RNA recognition motif (RRM) that consists of two sub-motifs, RNP1 and RNP2^16^. Recent reports have shown that RBM24 can post-transcriptionally regulate the stability of transcriptional factor p53 family members, such as p63 mRNA and p21 mRNA, through its RRM^16, 17^, further suggesting the importance of RBM24 in RNA biology. Data from our group indicate that RBM24 can modulate hepatitis C virus (HCV) translation and replication, implying that RBM24 may participate in viral replication^18^. Considering that HBV transcripts are typical mRNAs produced by host RNA polymerase II^19^, in which pgRNA is an essential replication intermediate, it is possible that RBM24 is involved in HBV replication via interacting with these viral transcripts. To test this hypothesis, we analyzed the function of RBM24 in the HBV life cycle. The data presented herein demonstrate that RBM24 is involved in HBV transcription and translation by binding to the terminal redundancy (TR) at the 3′ and 5′ termini of 3.5-kb HBV RNA through its RNA-binding domains. On the one hand, the interaction of RBM24 with the 3′ TR of HBV RNA stabilizes the post-transcriptional level of HBV RNA. On the other hand, the interaction of RBM24 with 5′ TR of HBV pgRNA hinders the translation of pgRNA into the core protein. The dual function of RBM24 suggests that RBM24 is a critical host gene that contributes to maintaining a balance between mRNA stability and translation.

## Results

### RBM24 is required for HBV replication

**Fig. 1:**
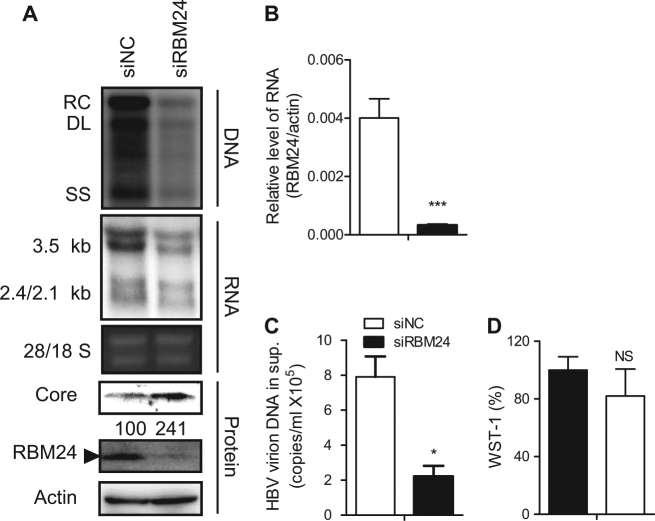
Knockdown of RBM24 impairs HBV replication and transcription. HepG2.2.15 cells were transfected with the indicated siRNA. **a** HBV replication intermediates were detected by southern blotting. The positions of relaxed circular (RC), double-stranded linear (DL), and single-stranded (SS) DNA are indicated (top panel). HBV transcripts were detected by northern blotting. Ribosomal RNA (28S and 18S) are presented as loading controls. The positions of HBV 3.5-kb, 2.4-kb, and 2.1-kb RNA are indicated (middle panel). RBM24 and core were detected by western blotting using an anti-RBM24 antibody or an anti-core antibody. The levels of β-actin served as a loading control (bottom panel). **b** The relative level of RBM24 was detected by real-time PCR. **c** HBV virions isolated from supernatants were used to quantify HBV DNA. **d** The WST-1 was detected to indicate the cytotoxic activities

To reveal the function of RBM24 in HBV replication, HepG2.2.15 cells, which contain an integrated HBV (subtype *ayw*) genome and stably express HBV driven by endogenous HBV promoters and enhancer elements, were transfected with RBM24-specific small interfering RNA (siRNA)^18^ or nonspecific siNC. Knockdown of RBM24 led to a significant reduction in HBV DNA and all transcripts, including the 3.5-kb viral pgRNA, compared with siNC-transfected cells (Fig. 1a, b). Correspondingly, HBV virions secreted into the supernatants (Fig. 1c) were also significantly decreased in siRBM24-transfected cells. Surprisingly, the core protein level was increased (Fig. 1a). These results indicated that RBM24 was involved in HBV replication.

### Ectopic expression of RBM24 suppresses HBV replication

**Fig. 2:**
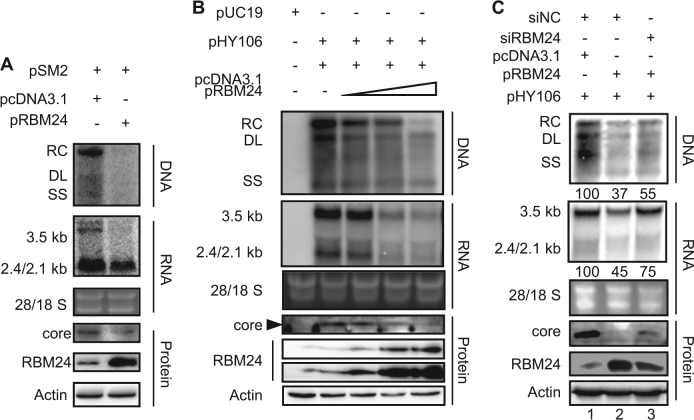
Ectopic expression of RBM24 suppressed HBV transcription and replication. **a** HepG2 cell were co-transfected with 1.5 μg of pSM2 and 0.5 μg of pRBM24 or empty vector in 6-well plates. **b** HepG2 cells were co-transfected with 0.8 μg of pHY106 and 0, 0.01, 0.1, or 0.5 μg of pRBM24 or empty vector in 6-well plates. **c** HepG2 cells were co-transfected with 0.8 μg of pHY106, 0.5 μg of pRBM24, and the indicated siRNA or empty vector in 6-well plates. **a–c** HBV replication intermediates were detected by southern blotting. The positions of relaxed circular (RC), double-stranded linear (DL), and single-stranded (SS) DNA are indicated (top panel). HBV transcripts were detected by northern blotting. Ribosomal RNA (28S and 18S) are presented as loading controls. The positions of the HBV 3.5-kb, 2.4-kb, and 2.1-kb RNA are indicated (middle panel). RBM24 and core were detected by western blotting using an anti-RBM24 antibody or an anti-core antibody. The levels of β-actin served as a loading control (bottom panel). Hybridization signals were quantified with NIH ImageJ software

To further validate the effect of RBM24 on HBV transcription and replication, HepG2 cells were co-transfected with pSM2 (a tandem dimer of HBV genome of genotype D) and the pRBM24 or empty vector. Intriguingly, we observed that overexpression of RBM24 also decreased the amount of HBV DNA, RNA (Fig. 2a). Simultaneously, the core protein level was decreased (Fig. 2a). Given that HBV transcription from pSM2 was controlled by the HBV promoter itself, pHY106, a 1.1-mer construct with pgRNA driven by the cytomegalovirus (CMV) promoter, was used to verify the effect of RBM24 overexpression on HBV replication. HepG2 cells were co-transfected with pHY106 and increasing concentrations of the pRBM24 or empty vector. As shown in Fig. 2b, overexpression of RBM24 also decreased the amount of HBV DNA, RNA, and the core protein in a dose-dependent manner. Therefore, RBM24 overexpression suppressed HBV replication. Considering that HBV transcription and replication in pHY106-transfected HepG2 cells were stronger than in pSM2-transfected cells, pHY106 was used in the subsequent study.

The observation that both knockdown and overexpression of RBM24 suppressed HBV replication and transcription implies that a proper level of RBM24 may be required for HBV replication. To address this issue, pHY106, pRBM24, or empty vector, and RBM24-specific siRNA or nonspecific siNC, were co-transfected into the HepG2 cell line. The overexpression of RBM24 decreased the amount of HBV DNA, RNA and the core protein level (Fig. 2c, lane 2). However, when siRBM24 was co-transfected, the levels of HBV DNA, RNA and core protein were partly rescued, along with a decrease in RBM24 levels (Fig. 2c, lane 3).

### The RNP domains of RBM24 interact with HBV RNA and are required for its participation in HBV replication

**Fig. 3:**
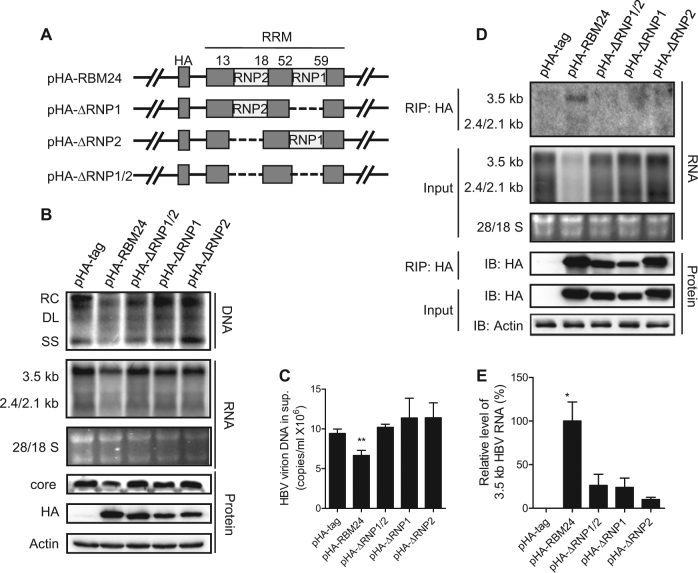
The RNP domains of RBM24 interact with HBV RNA, which are required for its participation in HBV replication. **a** Schematic illustration of the construction strategy of RBM24 deletion clones. **b**, **c** HepG2 cells were co-transfected with 0.8 μg of pHY106 and 0.5 μg of pHA-RBM24, pHA-ΔRNP1/2, pHA-ΔRNP1, pHA-ΔRNP2, or empty vector in 6-well plates. **b** HBV replication intermediates were detected by southern blotting. HBV transcripts were detected by northern blotting. RBM24 and core were detected by western blotting. **c** HBV virions isolated from supernatants were used to quantify HBV DNA. **d**, **e** HEK293T cells were co-transfected with 10 μg of pHY106 and 8 μg of pHA-RBM24, pHA-ΔRNP1/2, pHA-ΔRNP1, pHA-ΔRNP2, or empty vector in 100-mm dishes. Cell lysates were collected at 48 h post-transfection. RNA-IP assays were performed using anti-HA antibody. **d** The input or co-immunoprecipitated RNA and protein were detected. **e** The co-immunoprecipitated HBV 3.5-kb RNA was detected by real-time PCR

Previous reports have shown that RBM24 mainly functions via binding to RNA through its two putative RNA-binding sub-domains, RNP1 and RNP2^16, 17^. Three mutant forms of RBM24 lacking RNP1/2, RNP1, or RNP2 (ΔRNP1/2, ΔRNP1, or ΔRNP2) were generated (Fig. 3a) and co-transfected into HepG2 cells with pHY106. As shown in Fig. 3b, compared with the wild-type RBM24-transfected group, all mutant RBM24 proteins carrying a deletion of one (ΔRNP1 or ΔRNP2) or both RNP domains (ΔRNP1/2) showed no effect on the levels of HBV viral DNA, RNA (Fig. 3b), secreted virions (Fig. 3c), or core protein (Fig. 3b). These results confirmed that both the RNP1 and RNP2 domains were essential for the involvement of RBM24 in HBV replication.

Considering that both RNP1 and RNP2 are the RNA recognition domains (RRMs) and RBM24 lacking them had no influence on HBV, we speculated that RBM24 might interact with HBV RNA through its RNP domains. To test this hypothesis, an RNA-IP assay was performed. As shown in Fig. 3d, 3.5-kb and 2.4/2.1-kb RNA were detected only in precipitates expressing wild-type RBM24, but not in cells expressing any mutant form of RBM24 (ΔRNP1, ΔRNP2, or ΔRNP1/2), suggesting the specific association of wild-type RBM24 with HBV RNA (Fig. 3d). Notably, the 3.5-kb RNA signal was much stronger than the 2.4/2.1-kb RNA bands (Fig. 3d), implying a stronger interaction between RBM24 and 3.5-kb of RNA compared with the interaction between RBM24 and 2.4/2.1-kb of RNA. To validate the interaction between RBM24 and HBV RNA, real-time quantitative reverse transcription PCR was performed with primers specific to pgRNA (Fig. 3e). Compared with wild-type RBM24, the level of 3.5-kb RNA immunoprecipitated by mutant RBM24 ΔRNP1/2, ΔRNP1, or ΔRNP2 decreased to 26.3%, 24.0%, and 10.0%, respectively. These results indicated that 3.5-kb RNA mainly interacted with the RNP domains of RBM24. Taken together, these results indicated that RBM24 interacted with HBV RNA and that this interaction was mainly dependent on its RNP domains.

### TR fragments of HBV RNA interact with RNP domains of RBM24

**Fig. 4:**
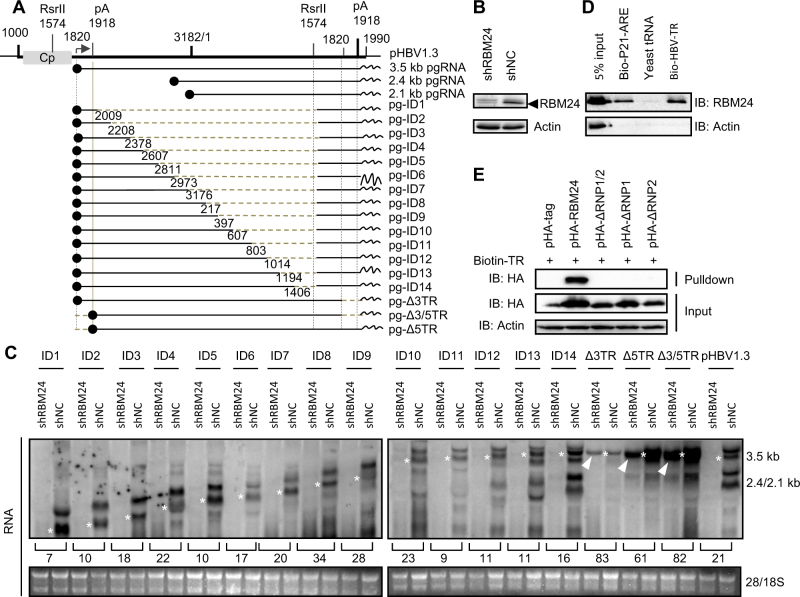
TR fragments of HBV RNA interact with RNP domains of RBM24. **a** Schematic illustration of the construction strategy of HBV deletion clones. **b** The level of RBM24 in the HepG2-shNC cells and HepG2-shRBM24 cells was detected by western blotting. **c** HepG2-shNC cells and HepG2-shRBM24 cells were transfected with HBV deletion clones. The HBV mRNA transcribed from the internal deletion clones was detected by northern blotting. The full-length or truncated pgRNA are indicated by “*”. **d** RBM24 cell lysates were pulled down with biotin-HBV-TR, biotin-p21-ARE (positive control), or yeast tRNA (negative control). **e** pHA-tag, pHA-RBM24, pHA-ΔRNP1/2, pHA-ΔRNP1, or pHA-ΔRNP2 cell lysates were pulled down with biotin-HBV-TR, and the input actin, HA-tag, HA-RBM24, HA-ΔRNP1/2, HA-ΔRNP1, and HA-ΔRNP2 served as controls

Subsequently, we further mapped the HBV RNA region(s) that interact with RBM24. A series of deletion clones of the HBV genome previously constructed to express HBV RNA fragments (Fig. 4a)^20^ were transfected into HepG2-shNC or HepG2-shRBM24 cells. Knockdown of RBM24 (Fig. 4b) clearly down-regulated the level of HBV RNA transcribed from the whole viral genome (pHBV1.3) (Fig. 4c). Similarly, the levels of RNA fragments expressed by the viral genome with consecutive internal deletions (nts 2009–3182/1–1574) were also obviously down-regulated, although the RNA levels varied among different constructs (Fig. 4c). Interestingly, when the TR (nts 1820–1918) was removed from the 3′ terminus (pg-Δ3TR) and/or the 5′ terminus (pg-Δ5TR, pg-Δ3/5TR) of pgRNA, the truncated pgRNA levels were only slightly down-regulated by knockdown of RBM24 (Fig. 4c, as indicated by the white arrow). These results indicated that both 5′ and 3′ TRs were important viral elements that responded to RBM24 and might therefore interact with RBM24.

To further validate the interaction between RBM24 and TR sequences of HBV RNA, a biotin pulldown assay was performed. The known binding partner of RBM24, the AU-rich element within the 3′ UTR of p21 mRNA^16^, was utilized as a positive control. Yeast tRNA without biotin labeling served as the negative control. Compared with the positive control, a stronger band corresponding to RBM24 was detected following addition of the HBV-TR fragment (Fig. 4d), suggesting that RBM24 interacted with the TR sequences of HBV RNA.

Based on this result, we further verified whether the RNP domains of RBM24 were essential for the interaction between TR sequences of HBV RNA and RBM24. As shown in Fig. 4e, only wild-type RBM24 was pulled down by HBV-TR fragments. Mutant RBM24 with deletions of any RNP domain was not pulled down by TR fragments, indicating that both RNP1 and RNP2 domains were required for the interaction of RBM24 with TR fragments of HBV RNA.

### RBM24 promotes stability of HBV RNA

**Fig. 5:**
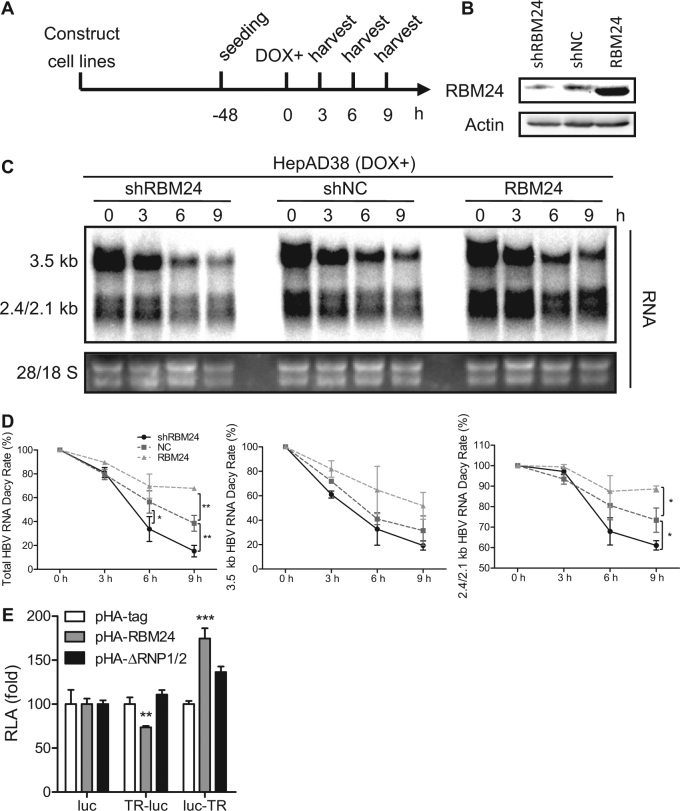
RBM24 promotes stability of HBV RNA. **a** HepAD38-shRBM24, HepAD38-shNC, and HepAD38-RBM24 cells were constructed and seeded at −48 h. DOX was added to the culture medium to shut down HBV RNA transcription at 0 h, and cells were harvested at the indicated time points. **b** The expression of RBM24 in cell lines was detected by western blotting. **c** HBV RNA was extracted from harvested cells and analyzed by northern blotting. **d** Kinetic analysis of HBV RNA decay in the cell lines. The relative levels of HBV RNA were normalized to 28S and expressed as the percentage of the RNA signals from the corresponding sample at time point 0 h. **e** HepG2 cells were co-transfected with each indicated reporter plasmid (pluc, pTR-luc, or pluc-TR) and control vector or plasmid expressing RBM24 or ΔRNP1/2. Cells were harvested at 48 hpt, and the relative luciferase activity (RLA) was measured

The 3′ TR fragment of HBV RNA is essential and important for maintaining the stability of HBV RNA^20–22^. The interaction of RBM24 with the 3′ TR fragment of HBV RNA suggested that RBM24 might regulate the stability of HBV RNA. To test this hypothesis, we analyzed the decay kinetics of HBV RNA during knockdown and overexpression of RBM24. The velocity of HBV RNA degradation was faster in HepAD38-shRBM24 but slower in HepAD38-RBM24 compared with the control HepAD38-shNC (Fig. 5b, c), indicating that RBM24 promoted the stability of HBV RNA. Further decay kinetics analysis showed that both 3.5-kb and 2.4/2.1-kb HBV RNA were stabilized by RBM24 (Fig. 5d).

Considering that the 3′ TR and 5′ TR in 3.5-kb HBV RNA have the same sequences^23^ and that our results also demonstrated RBM24 recognition of both 3′ TR and 5′ TR (Fig. 4c, d), to further confirm the functionality of the TR fragments, the TR region was inserted into plasmid pcDNA3.1-luc (pluc), either upstream or downstream of the luciferase gene, and a luciferase reporter assay was performed. Insertion of the TR downstream of the luciferase gene resulted in a significant up-regulation of luciferase activity in response to RBM24 expression, but not ΔRNP1/2 expression (Fig. 5e), supporting the conclusion that RBM24 increases RNA stability by targeting the 3′ TR of HBV RNA. However, the insertion of TR upstream of the luciferase gene resulted in a reduction of luciferase activity in response to RBM24 expression (Fig. 5e), suggesting that the interaction between RBM24 and the 5′ TR of 3.5-kb HBV RNA might serve different functions.

### RBM24 inhibits the translation of the core protein by binding to the 5′ TR of pgRNA and blocking 80S ribosome assembly on HBV pgRNA

**Fig. 6:**
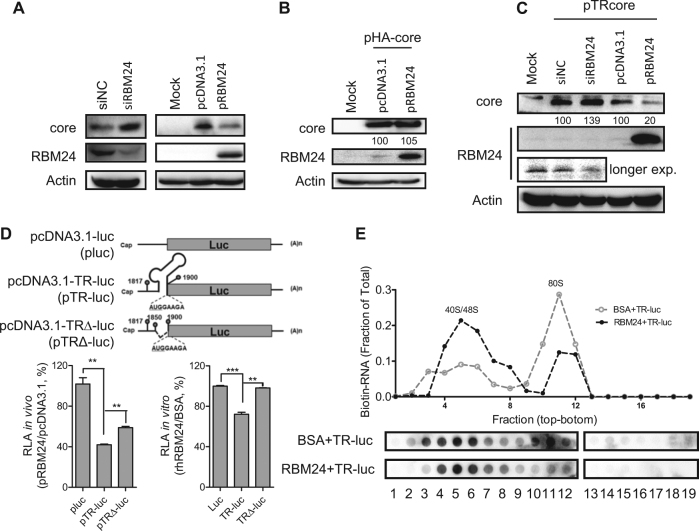
RBM24 inhibits the translation of the core protein by binding to the 5′ TR of pgRNA and blocking 80S ribosome assembly on HBV pgRNA. **a** The HepG2.2.15 cell line was transfected with siNC or siRBM24 (left panels), HepG2 cells were co-transfected with pHY106 and pRBM24 or empty vector (right panels). Cell lysates were collected at 48 hpt, and the expression of core and RBM24 was detected by western blotting. **b** HepG2 cells were co-transfected with pHA-core and pRBM24 or empty vector, and the expression of core and RBM24 was detected by western blotting. **c** HepG2 cells were co-transfected with pTR-core and the indicated siRNA or plasmid and harvested at 48 hpt. The expression of core and RBM24 was detected by western blotting. **d** HepG2 cells were transfected with the indicated plasmids, and luciferase activity was determined with Steady-Glo^®^. The relative luciferase activity (RLA) values were calculated and are shown in the bar graph on the left. The luciferase activity of 5′ TR-associated luciferase reporter plasmids in vitro was detected (bar graph on the right). **e** The 5′ TR-luciferase RNA together with rhRBM24 or BSA was incubated in rabbit reticulocyte lysate (RRL). The ribosome complexes were separated by sucrose gradient ultracentrifugation. The distribution of biotin-RNA was detected using a dot-blot assay

The 5′ TR fragment of HBV pgRNA is essential for the initiation of core protein translation from pgRNA^24^. Knockdown of RBM24 in HepG2.2.15 cells apparently enhanced the expression of the core (Figs. 1a and 6a). By contrast, overexpression of RBM24 in HepG2 cells impaired the expression of the core (Figs. 2a, b and 6a). However, co-transfection of pRBM24 and pHA-core plasmids with a CMV promoter did not affect the expression of core protein (Fig. 6b). These results implied that RBM24 was dependent on the 5′ TR of HBV RNA to inhibit translation of the core from pgRNA template. To further validate this speculation, the TR was inserted upstream of the core open reading frame (ORF) to generate the pcDNA3.1-TRcore (pTR-core) plasmid, which was then co-transfected with siNC, siRBM24, pcDNA3.1, or pRBM24, followed by the detection of core expression by western blotting. Knockdown of RBM24 increased while overexpression of RBM24 decreased core expression (Fig. 6c). These results further confirmed that RBM24 specifically affected the translation of core protein from pgRNA by targeting the 5′ TR region.

To further investigate whether the 5′ TR fragment of HBV pgRNA mediated the inhibition of core protein expression by RBM24, a series of expression plasmids containing a luciferase reporter gene were constructed as described in Materials and methods. Compared with the vector plasmid pluc, insertion of the 5′ TR of pgRNA (nts 1817–1903) containing the start codon of core (nts 1901–1903) between the T7 promoter and the luciferase ORF significantly reduced the relative luciferase activity (Fig. 6d). However, a partial deletion of the 5′ TR partly rescued the relative luciferase activity in vivo (Fig. 6d). These results suggested that RBM24 could inhibit core protein translation by interacting with the 5′ TR of the pgRNA. To further verify the impact of RBM24 on core protein translation, transcripts were generated in vitro and incubated with the recombinant human RBM24 protein (rhRBM24) protein or bovine serum albumin (BSA) in RRL. Like the in vivo assay results, the relative luciferase activity was significantly reduced by insertion of the 5′ TR of the pgRNA sequence, but was completely rescued by partial deletion of the 5′ TR stem loop of the pgRNA sequence (Fig. 6d). In addition, the results in Figs. 3d and 4e show that the RNP domains were essential for the binding of RBM24 to the 5′ TR of the pgRNA. Accordingly, overexpression of RBM24 with deletions in RNP1 and/or RNP2 did not affect the expression of the core protein (Fig. 3b). These results demonstrated that RBM24 inhibited the translation of core protein by binding to the 5′ TR of pgRNA.

To elucidate the mechanism underlying the inhibition of the 5′ TR-mediated translation activity by RBM24, ribosome assembly was examined in a rabbit reticulocyte lysate (RRL) system. Formation of the 80S ribosome was observed after incubation in the BSA control group, whereas the 80S peak was significantly less intense in the presence of rhRBM24 and most of the RNA was retained in the 40/48S peaks (Fig. 6e). Therefore, the inhibitory effect of the RBM24-5′ TR interaction on core protein translation was due to the inhibition of 80 S ribosome assembly on HBV pgRNA, occurring at the step after 40S ribosome binding.

These findings indicated that overexpression of RBM24 inhibited translation of the core and thus led to significant suppression of HBV replication.

### The impact of RBM24 on HBV replication in an in vitro infection system

**Fig. 7:**
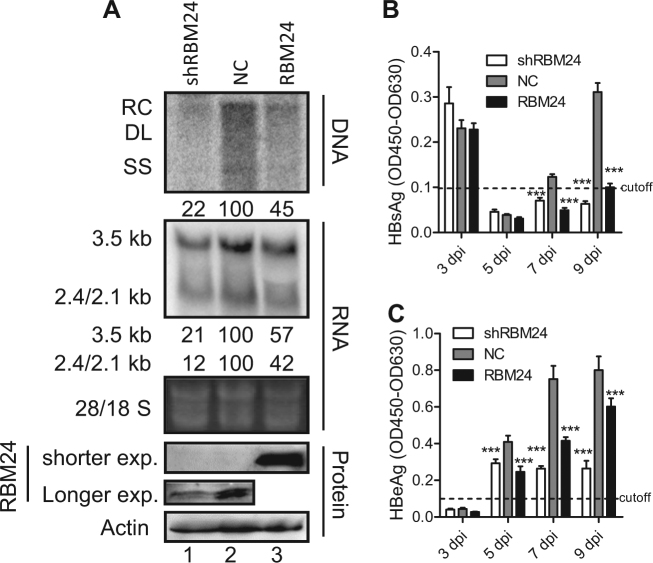
The impact of RBM24 on HBV replication and transcription in an in vitro infection system. Huh7-NTCP shNC, shRBM24, and RBM24 cells were seeded into 6-well plates and spinoculated with HBV virion particles. **a** HBV replication intermediates were detected by southern blotting. HBV transcripts were detected by northern blotting. The 28S and 18S fragments are presented as loading controls. RBM24 were detected by western blotting. Hybridization signals were quantified using the NIH ImageJ software. **b**, **c** HBsAg and HBeAg were detected by ELISA

Next, we validated the impact of RBM24 on HBV replication in an in vitro infection system. The Huh7-NTCP-shRBM24, Huh7-NTCP-NC, and Huh7-NTCP-RBM24 cell lines were constructed based on Huh7-NTCP followed by HBV infection. As shown in Fig. 7a, both knockdown and overexpression of RBM24 led to a significant reduction of HBV DNA and RNA, which was consistent with the results shown in Fig. 1 and Fig. 2. Correspondingly, the hepatitis B surface antigen (HBsAg) and hepatitis B e-antigen (HBeAg) levels in the supernatants were also significantly decreased in both Huh7-NTCP-shRBM24 and Huh7-NTCP-RBM24 cells (Fig. 7b, c). However, the core protein could not be detected by western blotting, potentially due to the low HBV infection efficiency.

## Discussion

Transcription of the HBV genome produces a viral pregenome and viral mRNA. Control of HBV at the level of transcription or post-transcription can orchestrate both HBV replication and gene expression. Host factors have been found to play an important role during this process^7–12^. In the present study, a novel host factor, RBM24, was identified to modulate the stability of HBV RNA at the post-transcription level, which was similar to some previously identified host factors^11, 20^. However, RBM24 could also inhibit the expression of core protein through interference with ribosome assembly and HBV RNA transcription.

In contrast to previously reported host factors^20, 25^ affecting HBV replication either positively or negatively, it seems that proper expression of RBM24 was important for HBV replication, as demonstrated by the knockdown data shown in Fig. 1 and further strengthened by the observation that transient co-transfection of both RBM24-specific siRNA and pRBM24 could partially rescue HBV replication and transcription (Fig. 2c). These results suggest that a proper level of RBM24 is required for HBV replication by regulating the balance between RNA stability and the core protein level. As RBM24 protein can bind to the 5′ and 3′ termini of HBV pgRNA, excessive amounts of RBM24 may occupy the binding sites and prevent the contact of both ends, which is considered to be essential for the HBV DNA replication process^26–29^. The exact stoichiometry of RBM24 binding to HBV pgRNA must be determined to fully explain these results.

The TR sequence of HBV RNA is composed of approximately 100 nt^23^. The 3′ TR contains sequences that are essential for correct and efficient polyadenylation^30^. In agreement with this finding, the interaction between RBM24 and the 3′ TR of HBV RNA did increase the stability of HBV RNA (Fig. 5). By contrast, for pgRNA, in addition to the 3′ TR, one copy of the TR site within the 5′ end of pgRNA^23^ plays key roles in viral translation, RNA packaging, and DNA priming^30^. Consistent with this point, the interaction between RBM24 and the 5′ TR of pgRNA inhibited the translation of pgRNA into the core protein (Fig. 6). Therefore, although the RBM24-3′TR interaction promoted the stability of pgRNA (Fig. 5), translation inhibition of the core protein due to the RBM24-5′ TR interaction caused the pgRNA to behave differently from the other HBV RNA. It is also conceivable that RBM24 binds more strongly to pgRNA than to other HBV RNA because pgRNA contains a 5′ TR in addition to a 3′ TR. Consistent with this speculation, a stronger signal corresponding to an mRNA of 3.5 kb was detected. By contrast, only a faint signal corresponding to an mRNA of 2.4/2.1 kb was detected (Fig. 3d). Thus, because deletion of the 3′ TR and 5′ TR did not completely abolish the impact of RBM24 on HBV (Fig. 4), we still could not rule out the possibility that RBM24 might interact with other fragments of HBV besides the 5′ TR and 3′ TR and thereby partly regulate viral transcription and replication. In addition, considering that the 5′ TR contains an important encapsidation signal, the ε element, the RBM24-5′ TR interaction might affect pgRNA packaging. Further studies are needed to clarify this issue.

It has been postulated that the translation of pgRNA into the core protein is initiated by ribosomal scanning, in which the 40S small ribosomal subunit binds to the 5′ cap structure, followed by linear scanning in the 3′ direction until it encounters the initiating AUG codon. Then, the 60S large subunit joins the complex to form a complete 80S ribosome, and translation is initiated^31^. As shown by both in vivo and in vitro assays, overexpression of RBM24 inhibited the translation of luciferase initiated by the 5′ TR, which contains an AUG codon. By contrast, the lack of a stem loop in the 5′ TR caused RBM24 to lose the action site, resulting in a subsequent loss of the inhibitory effect on the translation of luciferase (Fig. 6). Furthermore, a ribosome assembly assay showed that RBM24 did not affect the binding of the 40S subunit to mRNA containing a full-length 5′ TR, whereas it did affect the formation of the 80S ribosome assembly complex on mRNA containing a full-length 5′ TR. Thus, it is possible that RBM24 binds to the 5′ TR and thus blocks scanning of the 40S small ribosomal subunit to encounter the AUG codon, followed by failure of 80S ribosome assembly. Consequently, translation of the core protein was inhibited, which supports the idea that Pol may hamper ribosome assembly at the initiating AUG codon via the Pol-ε interaction, thus suppressing the translation of pgRNA into the core protein^24^. Therefore, overexpression of RBM24 inhibited the translation of the core, the assembly of nucleocapsids where HBV DNA synthesis occurs, and then suppressed HBV DNA replication.

We do not yet know how RBM24 regulates HBV RNA. RBPs are known to play a key role in the post-transcriptional regulation of different host genes, including mRNA stabilization and translation^32–34^. RBM24 also acts a transcriptional regulator by regulating transcription factors, such as p63 and p53^35, 36^. Hence, RBM24 might regulate some host transcriptional factor(s) that are involved in HBV transcription. We found that overexpression of RBM24 reduced the activities of four HBV promoters (Fig. S1). Considering that the comprehensive transactivator HBx could activate the CMV immediate early (CMV-IE) promoter^37–41^, it is possible that the down-regulation of HBx protein due to the inhibition of RBM24 overexpression on HBx promoter activity (Fig. S1) reduced CMV-IE promoter activity and thus led to a drastic reduction of HBV pgRNA. Therefore, overexpression of RBM24 reduced HBV RNA despite being driven by endogenous HBV promoters or by the CMV promoter (Fig. 2). Moreover, the reduction of HBV RNA and antigens by RBM24 overexpression in HBV-infected cells was likely due, at least in part, to the inhibition of viral promoter activity (Fig. 7). However, we also could not rule out other potential regulatory mechanisms. Further studies are needed to clarify this issue.

Taken together, our results identify a novel host factor, RBM24, that modulates the stability of HBV RNA at the level of post-transcription and expression of core protein at the level of ribosome assembly. Our work demonstrates that aberrant expression of RBM24 inhibits HBV replication via multiple mechanisms. This finding should provide insights into our understanding of the mechanism of HBV replication and the development of new antiviral targets.

## Materials and methods

### Cell cultures

HepG2, HepG2.2.15, HepAD38^42^, Huh7-NTCP (stable cell line expressing human NTCP was generated from Huh7 cells)^43, 44^, and human embryonic 293T (HEK293T) cells were cultured in Dulbecco’s modified Eagle’s medium supplemented with 2 mM glutamine (Gibco^®^, New York, NY, USA, 12100-046), 10% fetal bovine serum (Gibco^®^, Grand Island, NY, USA, 10099-141), and 100 U/ml penicillin–streptomycin (Gibco^®^, New York, NY, USA, 15140-122) at 37 °C in a 5% CO_2_ atmosphere. Plasmids and siRNA were transfected into HepG2 cells using Lipofectamine^®^ 3000 Reagent (Invitrogen™, Carlsbad, CA, USA, L3000-015) or HepG2.2.15, HepAD38, and HEK293T cells using Lipofectamine^®^ 2000 Reagent (Invitrogen™, Carlsbad, CA, USA, 11668-019) following the manufacturer’s instructions. The following siRNA were used: All Stars Negative Control siRNA (siNC, Qiagen, Hilden, Germany, SI03650318) and siRBM24 (Qiagen, Hilden, Germany, SI03030195). The cytotoxic activities were detected by WST-1 (Beyotime, Shanghai, China, C0036).

The plasmids pLKO.1-shNC, pLKO.1-shRBM24^16^, and pHAEG-RBM24 were constructed according to the manufacturer’s instructions. All the primers used in this study are listed in Table 1. In brief, Lenti-shNC, Lenti-shRBM24, and Lenti-RBM24 were produced by co-transfecting pSPAX2, pMD2G, and pLKO.1-shNC, pLKO.1-shRBM24, or pHAEG-RBM24 into HEK293T cells. The supernatants were collected after transfection, and the lentivirus stocks were aliquoted and stored at −80 °C. Cell lines were infected with the corresponding lentivirus to construct HepG2-shNC, HepG2-shRBM24, HepAD38-shNC, HepAD38-shRBM24, Huh7-NTCP-shNC, Huh7-NTCP-shRBM24, and Huh7-NTCP-RBM24 cell lines.

**Table 1:** Oligonucleotides used in this study

*Oligonucleotide*	*Sequence*
RBM24-SybrG-F	5′-GGCCAACGTGAACCTGGCATACTT-3'
RBM24-SybrG-R	5′-GGCAGGTATCCCGAAAGGTCTTTGT-3'
HBV-SybrG-F(1120)	5′-CACAACATCAGGATTCCTAGGACC-3′
HBV-SybrG-R(1120)	5′-TCCCCCTAGAAAATTGAGAGAAGTC-3′
pHA-core-F	5′-CCCAAGCTTGGGATGGACATTGACCCTTATAAAGAAT-3′
pHA-core-R	5′-CGGGGTACCGGGCTAACATTGAGATTCCCGAGATTGA-3′
p21-ARE-F	5′-TAATACGACTCACTATAGGGACTATCCGCCCACAGGAAGCCTGCAGTCCT-3′
p21-ARE-R	5′-AAGGAGAACACGGGATGCTTCCAGGACTGCAG-3′
pTR-core-F	5′-CCCAAGCTTGGGAACTTTTTCACCTCTGCCTAAT-3′
pTR-core-R	5′-CGGGGTACCCCGCTAACATTGAGATTCCCGAGATTGA-3′
HBV-1818-1935-F	5′-TAATACGACTCACTATAGGGACTAACTTTTTCACCTCTGCCTAATCAT-3'
HBV-1818-1935-R	5′-GTAGCTCCAAATTCTTTATAAGGGT-3'
shNC-F	5′-CCGGCCTAAGGTTAAGTCGCCCTCGCTCCTCGAGGCGAGGGCGACTTAACCTTAGGTTTTTG-3'
shNC-R	5′-AATTCAAAAACCTAAGGTTAAGTCGCCCTCGCTCCTCGAGGCGAGGGCGACTTAACCTTAGG-3'
shRBM24-UTR-F	5′-CCGGGCGAGCAATATGTAGCTTGAACTCGAGTTCAAGCTACATATTGCTCGCTTTTTG-3'
shRBM24-UTR-R	5′-AATTCAAAAAGCGAGCAATATGTAGCTTGAACTCGAGTTCAAGCTACATATTGCTCGC-3'
HR-RBM24-V-F	5′-CGGCGGCCGCACCGGTCTGCATGCACACGACCCAG-3'
HR-RBM24-V-R	5′-GGGAGGGATCCTCTAGACTCCTATTGCATTCGGTC-3'
luciferase-F	5′-GAAGACGCCAAAACATAAAGAAAGGC-3'
Luciferase-F-*Xba*1	5′-GCTCTAGAATGGAAGACGCCAAAAACA-3′
luciferase-R-*Hin*dIII	5′-CCCAAGCTTTTACACGGCGATCTTTCCGCCCTT-3'
TR-luc-F	5′-CTGTTCAAGCCTCCAAGCTGTGCCTTGGGTGGCTTTGGGGCATGGAAGACGCCAAAAACA-3'
TR△-luc-F	5′-GCTCTAGACAACTTTTTCACCTCTGCCTAATCATCTCTTGTAATGGAAGACGCCAAAAACA-3'
luc-TR-F	5′-CGGAAAGATCGCCGTGTAAAAGCTTCAACTTTTTCACCTCTGCCTAA-3′
luc-TR-R	5′-GGCTGATCAGCGGTTTAAACTTAAGGTAGCTCCAAATTCTTTATA-3′
3.5-kb HBV RNA-(2270–2288)-F	5′-GAGTGTGGATTCGCACTCC-3′
3.5-kb HBV RNA-(2270–2288)-R	5′-GAGGCGAGGGAGTTCTTCT-3′
RNP forward primer	5′-CCCAAGCTTATGCACACGACCCAGAAGGACACG-3′
RNP reverse primer	5′-GGGGTACCCTATTGCATTCGGTCTGTCTGCAG-3′
△RNP1 forward primer	5′-ACCGGCAGACGGGCAAGTCCGCTGACCGGGCTGCTGCCGAAAGGG-3′
△RNP1 reverse primer	5′-TCGGCAGCAGCCCGGTCAGCGGACTTGCCCGTCTGCCGGTCGGTG-3′
△RNP2 forward primer	5′-AGGACACGACGTACACCAAGCCCTACCACACCACCGACGCCAGCC-3′
△RNP2 reverse primer	5′-GCGTCGGTGGTGTGGTAGGGCTTGGTGTACGTCGTGTCCT-3′

### Plasmids

The HBV (genotype A, subtype *adw*2, GenBank accession number AF305422.1) replication-competent plasmid pHY106 is a 1.1-mer construct with pgRNA driven by the CMV promoter and has been described previously^45^. The pSM2 plasmid (genotype D) is a 2-mer construct driven by the HBV promoter itself and has been described previously^46^. To construct RBM24 protein expression plasmids, the ORF of human RBM24 was amplified from cDNA and cloned into pcDNA3.1 (Invitrogen, Carlsbad, CA, USA), pXJ40-Flag and pXJ40-HA (pHA-tag, Invitrogen, Carlsbad, CA, USA) backbones to generate pcDNA3.1-RBM24 (pRBM24), pXJ40-Flag-RBM24 (pFlag-RBM24), and pXJ40-HA-RBM24 (pHA-RBM24). Gene expression cassettes of RBM24 lacking RNP1 or RNP2^16^ were amplified and cloned into pXJ40-HA to generate pHA-RBM24ΔRNP1 (pHA-ΔRNP1), pHA-RBM24ΔRNP2 (pHA-ΔRNP2), and pHA-RBM24ΔRNP1/ΔRNP2 (pHA-ΔRNP1/2). HBV pgRNA internal deletion clones (pgID-1 to pgID14, pg-Δ3TR, pg-Δ5TR, pg-Δ3/5TR) and their wild-type control pHBV1.3 were kindly provided by Prof. Haitao Guo^20^. The ORF of HBV core protein was amplified from pHY106 and cloned into pXJ40-HA to generate pHA-core. The fragment containing the 5′ TR sequence and ORF of HBV core protein was amplified from pHY106 and cloned into pcDNA3.1 to generate pTR-core.

The ORF of luciferase was amplified from pGL2-base (Promega, Madison, WI, USA) and cloned into pcDNA3.1(−) to generate pcDNA3.1-Luc (pluc), an expression plasmid containing a luciferase (luc) reporter gene downstream of a T7 promoter. To obtain pcDNA3.1-TR-luc (pTR-luc) or pcDNA3.1-luc-TR (pluc-TR), the 5′ TR or 3′ TR of the HBV pgRNA sequence (nts 1818–1903) was fused to the luciferase ORF, and the AUG codon of luciferase was replaced with the AUG codon of the core (nts 1901–1903) so that the AUG codon (nts 1901–1903) could initiate the expression of luciferase. A portion of the 5′ TR sequence (nts 1850–1900) except for the AUG codon of the core (nts 1901–1903) was deleted from pcDNA3.1-TR-luc to generate pcDNA3.1-TRΔ-Luc (pTRΔ-luc). All the primers used in this study are listed in Table 1.

### HBV DNA analysis

HBV replication intermediates in transfected cells were extracted and subjected to southern blotting as described previously^47^. Hybridization signals were quantified with the NIH ImageJ software. In brief, to detect virion DNA in cell culture media, virions were immunoprecipitated using anti-BJ11 antibody, anti-S1 antibody, and anti-S39 antibody (kindly provided by Bing Yan); then, HBV virion DNA was quantified by real-time PCR.

### RNA analysis

Total RNA was extracted from cells using TRIzol reagent (Invitrogen, Carlsbad, CA, USA, 15596-018) as described previously^48^. Northern blotting and real-time PCR were performed to detect HBV RNA or RBM24 mRNA. Hybridization signals were quantified with the NIH ImageJ software.

### Western blotting

Western blotting analysis was performed as described previously^49, 50^. The following antibodies were used: anti-actin (catalog number sc-47778; Santa Cruz, Dallas, TX, USA), rabbit anti-HA (catalog number 3724S; Cell Signaling Technology, Danfoss, MA, USA), mouse anti-HA (catalog number H9658; Sigma, St. Louis, MO, USA), anti-RBM24 (catalog number ab94567; Abcam, Cambridge, UK), anti-HBc (catalog number GB058629; Gene Tech, Shanghai, China), streptavidin horse radish peroxidase (catalog number CT353; U-cytech, Utrecht, The Netherlands), anti-mouse secondary antibodies (catalog number 115-035-146; Jackson, Grand Island, NY, USA), anti-rabbit secondary antibodies (catalog number 111-035-003; Jackson, Grand Island, NY, USA), and Alexa Fluor 568 (catalog number A10042; Life Technologies, Carlsbad, CA, USA).

### Enzyme-linked immunosorbent assay

HBsAg and HBeAg in culture supernatants were detected as described previously^47^.

### RNA immunoprecipitation

RIP assays were performed using a previously described method^18, 51^. In brief, HEK293T cells were co-transfected with pHY106 and pHA-RBM24, pHA-ΔRNP1/2, pHA-ΔRNP1, or pHA-ΔRNP2, and cell lysates were harvested at 48 h post-transfection (hpt). The cell lysates containing 800 μg total protein were incubated with protein G precoated with mouse anti-HA (catalog number H9658; Sigma, St. Louis, MO, USA) at 30 °C for 4 h. After washing five times, the precipitated RNA was detected by real-time PCR and northern blotting.

### Streptavidin pulldown assay

The streptavidin pulldown assay was performed as described previously^18, 52^. In brief, HEK293T cells were transfected with the indicated plasmids, and cell lysates were harvested at 48 hpt. Biotin-labeled RNA was incubated with cell lysates followed by pulldown with Dynabeads M-280 Streptavidin (Invitrogen, Carlsbad, CA, USA, 11205D) and assessment by western blotting.

### Luciferase reporter assay

The luciferase reporter assay was performed as described previously^53^. In brief, HepG2 cells were co-transfected with the indicated plasmids. At 48 hpt, the firefly luciferase activities were measured with a luciferase reporter assay system (Promega, Madison, WI, USA, E2940). All reporter assays were repeated at least three times.

### In vitro translation and ribosome assembly assay

Capped RNA from pluc, pTR-luc, or pTRΔ-luc was generated by in vitro transcription with a mMESSAGE mMACHINE^®^ Kit (Invitrogen, Carlsbad, CA, USA, AM1344). Biotin-11-UTP (Invitrogen, Carlsbad, CA, USA, AM8450) was added to the reaction to produce biotin-labeled capped RNA. The in vitro translation and ribosome assembly assays were performed as described previously^54^. In brief, template RNA and rhRBM24^18^ or a nonspecific control protein, BSA, were incubated in an RRL (Promega, Madison, WI, USA, L4960)-based translation reaction system followed by a luciferase activity assay with Steady-Glo^®^ (Promega, Madison, WI, USA, E2520)^53^.

### HBV infection and immunofluorescence

A Huh7-NTCP stable cell line expressing human NTCP was generated from Huh7 cells^43, 44^. Huh7-NTCP shNC, shRBM24, and RBM24 cells were spinoculated with HBV virion particles derived from HepAD38 cells at 1000 virus genome equivalents per cell, cellular supernatants were collected at 3, 5, 7, and 9 days post-infection (dpi), and cell lysates were collected at 9 dpi.

### Statistical analysis

The data were analyzed using a two-tailed unpaired *t* test. Statistical significance was set at “NS”*p* > 0.05, *p < 0.05, **p < 0.01, or ***p < 0.001.

## Electronic supplementary material

Figure S1
