# Supplementary material for: RBM24 stabilizes hepatitis B virus pregenomic RNA but inhibits core protein translation by targeting the terminal redundancy sequence
Source: Emerg Microbes Infect. 2018 May 14;7:86. doi: 10.1038/s41426-018-0091-4 (PMC5951808; doi:10.1038/s41426-018-0091-4)
Supplement: Supplementary file 1 — Figure S1 [file 41426_2018_91_MOESM1_ESM.pptx]

## Slide 1
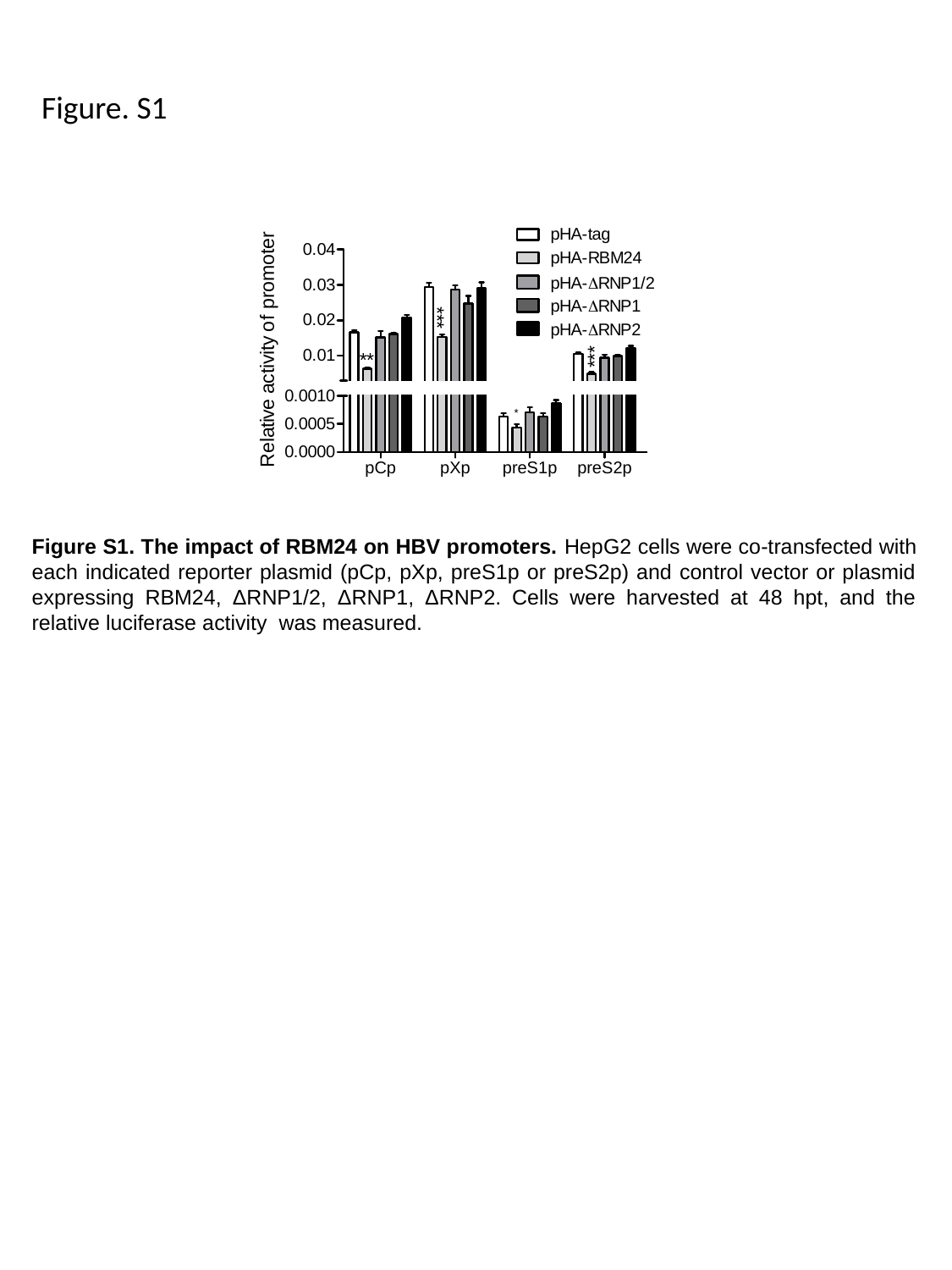

Figure. S1
Figure S1. The impact of RBM24 on HBV promoters. HepG2 cells were co-transfected with each indicated reporter plasmid (pCp, pXp, preS1p or preS2p) and control vector or plasmid expressing RBM24, ΔRNP1/2, ΔRNP1, ΔRNP2. Cells were harvested at 48 hpt, and the relative luciferase activity was measured.
